# A simple and fast method for estimating bat roost locations

**DOI:** 10.1098/rsos.231999

**Published:** 2024-04-24

**Authors:** Lucy Henley, Domhnall Finch, Fiona Mathews, Owen Jones, Thomas E. Woolley

**Affiliations:** ^1^ Cardiff School of Mathematics, Cardiff University, Cardiff CF24 4AG, UK; ^2^ University of Sussex, John Maynard Smith Building, Brighton BN1 9RH, UK; ^3^ National Parks and Wildlife Service, North Dublin D07 N7CV, Ireland

**Keywords:** bat movement, roost finding, diffusion, partial differential equation model, ecology

## Abstract

Bats play a pivotal role in pest control, pollination and seed dispersal. Despite their ecological significance, locating bat roosts remains a challenging task for ecologists. Traditional field surveys are time-consuming, expensive and may disturb sensitive bat populations. In this article, we combine data from static audio detectors with a bat movement model to facilitate the detection of bat roosts. Crucially, our technique not only provides a point prediction for the most likely location of a bat roost, but because of the algorithm’s speed, it can be applied over an entire landscape, resulting in a likelihood map, which provides optimal searching regions. To illustrate the success of the algorithm and highlight limitations, we apply our technique to greater horseshoe bat (*Rhinolophus ferrumequinum*) acoustic data acquired from six surveys from four different UK locations and over six different times in the year. Furthermore, we investigate what happens to the accuracy of our predictions in the case that the roost is not contained within the area spanned by the detectors. This innovative approach to searching rural environments holds the potential to greatly reduce the labour required for roost finding, and, hence, enhance the conservation efforts of bat populations and their habitats.

## 1. Introduction

Bats are a diverse group of flying mammals, comprising over 1000 species worldwide [1]. They play crucial ecological roles globally across terrestrial ecosystems as they contribute to pest control, pollination and seed dispersal [2,3]. Despite their ecological importance, locating their roosts is a challenge. Thus, the conservation and study of bats have been hampered by their elusive nature [4].

Bat roosts are essential to their survival and reproductive success, making them focal points for ecological research and conservation [5]. The traditional methods for identifying bat roosts, such as field surveys and visual inspections of potential roosting sites, are not only labour-intensive but also often disturb the sensitive bat populations they intend to study [6]. Furthermore, the cryptic behaviour and nocturnal habits of bats compound the difficulties associated with their roost detection.

Critically, bats are highly susceptible to anthropogenic disturbances due to their sensitivity to changes in lighting, noise, land use and climate, among other factors. Furthermore, the effects of habitat fragmentation resulting from urban development can significantly diminish foraging opportunities, which poses a substantial risk to bat populations [7].

In the summer maternity season, female bats aggregate to give birth and rear their young. Consequently, the identification and protection of maternity roosts are of paramount importance for bat conservation efforts. Disturbance to bat roosts has been recognized as a key driver of population declines in bat species across Europe over the past century [8,9]. As a response to these conservation concerns, bats are protected by law in many countries [10–12].

In recent years, there has been growing interest in exploring non-invasive methods to identify and monitor bat roosts that reduce ecological disturbance while increasing detection efficiency [13]. The integration of mathematical techniques and advanced analytical tools has emerged as a promising avenue for addressing these challenges.

An approach to locating birds’ nests from GPS tracking surveys that result in similar data to that of radio-tracking surveys has been implemented in the R package NestR [14]. The package uses recursive movement patterns (periodic returns to places of ecological significance) [15] to identify the locations of ecologically relevant locations such as nests.

Alternatively, geographic profiling is a method commonly used in criminology to determine the most probable area of an offender’s residence [16]. It is used in cases of serial-linked crimes and assumes that crimes are most likely to occur close to the offender’s home. In the case of locating a bat roost, a jeopardy surface (a surface representing the probability of a roost being located on each grid square) is produced using the locations of known foraging sites and the distance to each grid square. These techniques have been successfully used, along with radio-tracking studies, to narrow down search areas for roosts of pipistrelle bats [17].

In this article, we build on previous work, which used radio-tracked bat trajectories to extract movement features [18,19]. Using a model of diffusive agents, we can theoretically calculate how many bat passes should occur at different points over a given terrain, which can be directly compared with data taken from acoustic detectors spread around the environment, which detect the echolocation calls of individual bats as they travel by.

Due to this process being parallelizable, we can rapidly evaluate the difference between theory and data at all locations in the environment. The minimum difference indicates the most likely point that the roost inhabits, but, as we will see, the entire error surface provides crucial information regarding the accuracy of prediction.

This approach will be compared with an even simpler metric, which we will call the centre of calls (CC). This roost prediction point is defined to be the weighted average of detector positions, where the weights are the proportion of calls recorded at each detector. In the current work, we will see that CC does a very good job of predicting the roost location. However, it is limited to cases where the detectors surround the roost, thus, we extend our investigations to consider a case where the roost is not contained in the extents of the detector locations.

We begin in §2, where we introduce and discuss the data we will be using throughout the article to motivate and evaluate our roost searching algorithm. In §3, we develop our mathematical model and searching algorithm, which is then applied to datasets in §4. Since all our datasets contain the roost’s location within the extent of the detector placements, we take a brief diversion in §5 to consider the possibility of datasets that do not surround a roost. Finally, in §6, we discuss many of the algorithm’s limitations and underlying assumptions, resulting in a discussion on future developmental directions that could potentially increase the accuracy of our searching algorithm.

## 2. Static detector surveys

Many bats use echolocation to navigate and catch prey [20]. As such, acoustic surveys using bat detectors have proven to be a useful and cost-effective method to study populations [21,22].

Passive acoustic bat detectors are recording devices calibrated to record the high-frequency sound of bat calls and allow for long-term, autonomous surveys. The detection distance varies according to species and habitat, but often ranges from 10 to 30 m, with greater horseshoe bats (*Rhinolophus ferrumequinum*) being at the lower end of this range.

Surveys are conducted by placing detectors within a search area. The detectors then record the calls of bats passing through their range. The coordinates of each detector are noted and a series of recordings is produced, providing a spatio-temporal map of bat presence. Furthermore, the species present in each recording can be identified (either manually or automatically) in most cases through using features from the recordings, such as repetition rate, peak amplitude and shape of the waveform of the call [23,24].

In this work, we will use call data from only the first 90 min after sunset, which corresponds to the time of peak activity levels for greater horseshoe bats, and previous work has shown that the movement during this time correlates to a diffusive spreading of the bats finding foraging grounds. After this time, the bat population begins a slow return to the roost, and the movement can no longer be considered diffusive [18,19]. While there is often a second peak in activity levels close to sunrise, this is not always the case for all species; for example, two species of pipistrelle (the two most common bat species in the UK), have been shown to have only a single peak in activity levels, just after sunset [25]. As a result, we do not consider the activity levels beyond the first 90 min after sunset, as this would probably not be generalizable to other species.

In this article, we will be working with location data specified in terms of ‘eastings’ and ‘northings’. Eastings and northings are a coordinate system used in the UK that is based on the Ordnance Survey National Grid [26]. As is standard in mathematics, the coordinates are reported in the form (eastings, northings), corresponding to the idea that eastings are coordinates along an x -axis and northings are coordinates along a *y*-axis. If the grid coordinates are not given in parentheses, like Cartesian coordinates, then it is assumed that they are reported as eastings first and northings second. Effectively, eastings and northings are a metric Cartesian coordinate system measuring the distance in metres relative to a point located off the southwestern coast of the UK, in the Atlantic Ocean, near the Isles of Scilly. Specifically, the exact origin of the eastings and northings grid is given by the decimal latitude and longitude coordinates (49.766809 and –7.5571598). Although the choice of this National Grid Origin is arbitrary, the given point was selected to ensure that all coordinates in the UK would have positive values and, as the name suggests, increases in an x or *y* coordinate corresponds to a movement in an easterly or northern direction, respectively.

Having defined our coordinate system, we can specify the positions of the detectors and roosts. Firstly, in the case of the six surveys, we are going to assume that there is one roost, the location of which is known and defined to be $z_{R}$ . Furthermore, suppose there are N detectors, which are labelled i=1,…,N . The position of each detector is $X_{i}=(x_{i},y_{i})\in R^{2}$ . We can use our survey data to calculate the average number of calls per day at each detector site i , denoted $c_{i}$ . In the current case, as shown in table 1, bat counts have been done at each of the roosts of interest. However, generally, we are unlikely to know the exact number of bats in the colony. Thus, although it is possible to estimate the number of bats in a colony using static audio detectors [27], we will demonstrate that our method does not require this knowledge as we normalize the number of calls per day at each location to be a proportion of the total

**Table 1.** Details of each of the six surveys at Buckfastleigh, Braunton, Gunnislake and High Marks Barn.

roost	manual bat count	acoustic survey start date	surveyed area (km^2^ to 2 d.p.)	number of detectors	length of survey (days)	total number of detections	detections between sunset and 90 min after
Buckfastleigh	1798	26 Jun 2016	15.13	21	7	1458	567
Braunton	512	11 Jul 2016	15.56	19	7	793	357
Buckfastleigh	1798	25 Jul 2016	9.57	26	8	2728	698
Gunnislake	252	8 Aug 2016	12.24	21	7	270	112
High Marks Barn	746	22 Aug 2016	16.60	31	7	725	272
Buckfastleigh	1798	5 Sep 2016	26.23	21	8	441	115


(2.1) $$C_{i}=\frac{c_{i}}{\sum_{i=1}^{N}c_{i}}.$$

We will look at the results of six acoustic surveys of greater horseshoe bats conducted at four different UK maternity roosts (used between May and September by females and their young) in Devon throughout the summer of 2016 [28]. The surveys were conducted at Buckfastleigh (June, July and August), Braunton (July), Gunnislake (August) and High Marks Barn (August). See table 1 for further survey details. All surveys were originally conducted to study how bats use the landscape surrounding the roost, and the impact of different terrain types on foraging behaviour [29,30].

Manual counts were taken at each roost throughout the summer of 2016, and the number of bats found in each roost is shown in table 1. The counts were obtained in July, with multiple observers being positioned by exits from the roosts and counting the bats as they emerged at dusk. All four of the roosts specified in table 1 are large, used by hundreds of bats during the maternity season; however, the Buckfastleigh roost is by far the largest and is thought to be one of the largest greater horseshoe roosts in Europe, used by approximately 1800 bats during the maternity season of 2016.

**Figure 1.**
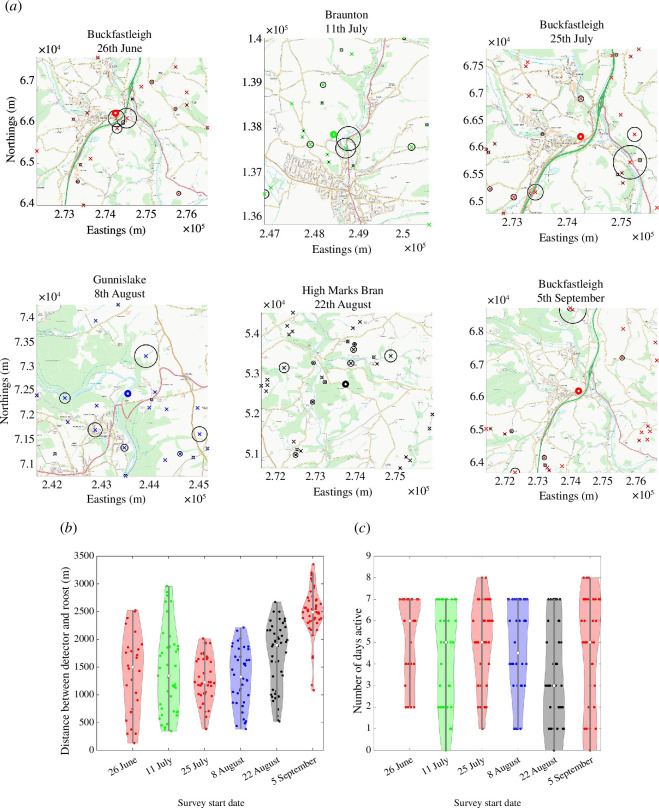
Details of each of the six surveys at Buckfastleigh (red), Braunton (green), Gunnislake (blue) and High Marks Barn (black). (*a*) Absolute positions of the detectors ( × marks) and roosts ( ∘ mark) at each surveyed location. The positions are in terms of eastings and northings. The background image illustrates the Ordinance Survey map, which highlights environmental use in terms of urban, suburban, road and river coverage. The black circle around each detector represents the relative proportion of calls. Specifically, the radius of the circle around detector i is $C_{i}$ km. (*b*, *c*) Violin plots illustrating various statistics regarding the data. The width of each violin plot is scaled by the percentage of records at each value on the *y*-axis. (*b*) The distribution of distances from each detector to the roost (as the crow flies). (*c*) Distributions of the number of nights that detectors were active.

The roost sites were known beforehand, and detectors were placed as evenly as possible around the roost location, given constraints caused by access issues, while trying to cover a variety of different terrain types [28]. The detector locations, land use and relative proportion of calls are illustrated in figure 1*a*.

The distribution of distances between each detector and the roost for each survey is shown in figure 1*b*. The minimum distance between detectors and the roost is seen in the first Buckfastleigh survey, at a separation of 130 m, while detectors are placed significantly further away in the last Buckfastleigh survey, with the closest placed 1.1 km away.

Since we require the activity of an average night we need to average the number of calls over the total number of nights that each detector was active. Unfortunately, due to technical faults, not all detectors ran for the entire duration of the survey. The number of nights over which detectors were active for each of the six surveys is shown in figure 1*c*. All detectors were active for between 0 and 8 nights before either the survey ended or the detector malfunctioned. The mean number of active nights for each survey ranges between 3 and 6.

In this section, we have introduced the data and defined our coordinate system. Next, we will use diffusion as a model of bat motion to estimate the number of expected calls at each detector, given a simulated roost location.

## 3. Adapting the diffusion model

As was shown in [18,19], greater horseshoe bat movement for the first 90 min after sunset can be modelled as diffusive. We will use a deterministic partial differential equation (PDE) diffusion model to estimate the expected number of calls at each detector using a predicted roost location, z , as a parameter to be estimated.

In this case, a deterministic model is preferable to a stochastic model, as simulating stochastic data is significantly more computationally expensive [31]. Critically, we will demonstrate how we can approximate the analytical solution to the diffusion PDE, which will provide an algebraic formula for the proportion of bat calls at each detector site. The simplicity of evaluating a formula, rather than simulating stochastic diffusing agents provides huge time saving [32]. Such gains in efficiency are important because the model has to be evaluated many thousands of times over an entire landscape.

Critically, we note that the deterministic equations provide excellent comparisons with the mean average behaviour of the stochastic simulations when the number of agents being simulated is large [33,34]. We will see *a posteriori* that the bat populations considered here which are of a few hundred to a few thousand are sufficient to produce good roost position estimates.

We assume that bats leave the roost at sunset and fly away from the roost in search of food. Foraging bats are modelled as diffusive particles on an infinite domain for the first 90 min after sunset [18,19]. Let $\phi (x,y,z_{x},z_{y},t)$ be the probability density of a bat’s location, (x,y) , at time t , given that the bat roost is at $\left(z_{x},z_{y}\right)$ then ϕ is defined by the PDE


(3.1) $$\begin{array}{r} \frac{\partial \phi (x,y,z_{x},z_{y},t)}{\partial t}=D\nabla_{\left(x,y\right)}^{2}\phi (x,y,z_{x},z_{y},t), \end{array}$$

(3.2) $$\begin{array}{r} \phi (x,y,z_{x},z_{y},0)=\delta^{2}(x-z_{x},y-z_{y}). \end{array}$$

where $\nabla_{\left(x,y\right)}^{2}$ is the Laplacian, which is just the linear combination of second derivatives with respect to the eastings and northings Cartesian coordinates, (x,y) . The parameter D is called the diffusion coefficient and it is a positive constant measuring the rate of spread of the bats.

We use the data calculated in our previous paper [19] using the relationship between mean squared distance travelled from the roost and time from sunset to provide a value of this parameter. Specifically, a typical value calculated from tracking surveys is $D\approx 80\;\;m^{2}s^{-1}$ .

The initial condition $\delta^{2}(x-z_{x},y-z_{y})=\delta (x-z_{x})\delta (y-z_{y})$ is the two-dimensional Dirac delta function [35], which specifies that all bats start the night at a roost located at $z=(z_{x},z_{y})$ . The model assumes infinite space, thus, we require that solutions satisfy the boundary conditions $\phi (x,y,z_{x},z_{y},t)\to 0$ , as |u|,|v|→∞ .

System (3.1) and (3.2) can be solved explicitly,


(3.3) $$\phi (x,y,z_{x},z_{y},t)=\frac{1}{4\pi Dt}\exp\left(-\frac{(x-z_{x}{)}^{2}+(y-z_{y}{)}^{2}}{4Dt}\right).$$

Although the movement of the diffusive agents is unbounded, equat[Disp-formula uFD4]ion (3.3) can be evaluated to show that the probability of a bat travelling further than m is typically small. Specifically, at time T=90 min, with a diffusion coefficient of $D=80\;m^{2}s^{-1}$ , the probability of a bat flying a distance beyond $R_{f}$ the roost is


(3.4) $$p\left(\sqrt{x^{2}+y^{2}}>R_{f}\right)=1-\int_{0}^{2\pi }\int_{0}^{3000}\phi (r\cos(\theta ),r\sin(\theta ),0,0,90\times 60)r\text{ d}r\text{ d}\theta <0.01,$$

or less than 1%. It is important to ensure that the probability of a bat being beyond $R_{f}$ at the end time is small, as most detectors are placed within a radius of 3 km of a suspected roost (see figure 1). Moreover, this was identified as the approximate radius of the core sustenance zone (CSZ) of the greater horseshoe bats. Namely, although the bats can fly further than the CSZ, they usually do not [18,36].

**Figure 2.**
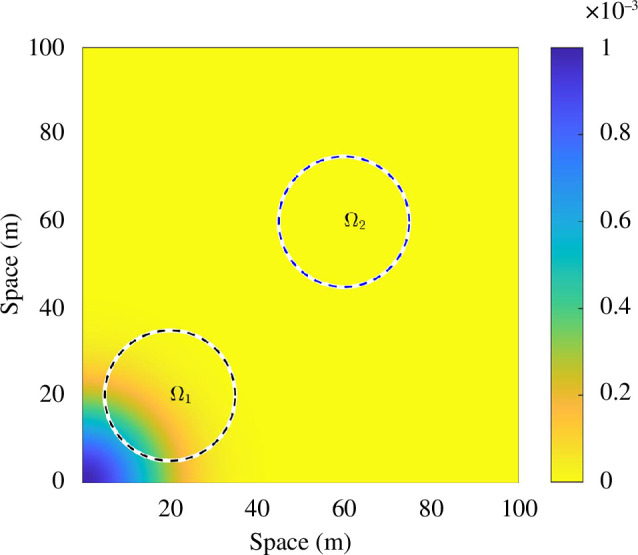
Illustrating the range of detection. The image shows the positive quadrant of ϕ(x,y,0,0,1) . The two circles illustrate two possible detection regions, $\Omega_{1}$ and $\Omega_{2}$ , where a detector has been placed at (20, 20) (black circle) and the other has been placed at (60, 60) (blue circle). Diffusion parameter is $D=80\;m^{2}\;s^{-1}$ .

Having generated a functional form of ϕ we can use equation (3.3) to estimate the proportion of calls that should be detected at each detector. Specifically, we assume that each detector i that is placed at a location $X_{i}=(x_{i},y_{i})$ has an active domain, $\Omega_{i}$ , in which it can detect a bat’s call. In our case, we assume that all microphones used in the surveys will register a bat call anywhere within a circle of radius 15 m about the detector’s location (see figure 2).

Integrating ϕ over a detector’s active region, $\Omega_{i}$ , provides the probability of the detector i detecting a bat within $\Omega_{i}$ at time t , given the roost was thought to be at $\left(z_{x},z_{y}\right)$ ,


(3.5) $$P_{i}(z_{x},z_{y},t)=\int_{\Omega_{i}}\phi (x,y,z_{x},z_{y},t)d\omega .$$

Thus, the expected number of calls between 0<t<T would be


(3.6) $$E_{i}(z_{x},z_{y})=\int_{0}^{T}P_{i}(z_{x},z_{y},t)n\text{ d}t,$$

where n is the number of bats leaving the roost. Finally, the proportion of calls registered at each detector (assuming a roost at $\left(z_{x},z_{y}\right)$ ) would be


(3.7) $$F_{i}(z_{x},z_{y})=\frac{E_{i}}{\sum_{j=1}^{N}E_{j}},$$

thus, n cancels out and we do not need to know how many bats there are in the roost.

Suppose we now consider a bat population with roost centred at the origin $\left(z_{x},z_{y})=(0,0\right)$ . Further, suppose there are two detection regions, $\Omega_{1}$ , centred at (20, 20) m and $\Omega_{2}$ , centred at (60, 60) m, illustrated in figure 2. Because $\Omega_{1}$ is nearer to the roost than $\Omega_{2}$ , we observe that the range of ϕ in $\Omega_{1}$ varies more than the range of ϕ in $\Omega_{2}$ ; ϕ is practically constant over $\Omega_{2}$ . Using this observation, we can approximate the integral that needs to be evaluated to generate the proportion of bat calls at each location and convert it into a simple algebraic formula. This observation is key to the simplification; namely, the simplification does not work unless the density distribution rapidly decays away from the source. Although this assumption holds in the current situation where we are modelling the bat spread as an evolving Gaussian distribution, which has been seen to be appropriate, we should be highly critical of this assumption if our model is to be applied to any other situation. In alternative applications, where the density distribution is known but is not rapidly decaying, then we could evaluate the integral, which is not difficult to simulate. However, the integral evaluations would cost more time than evaluating the algebraic approximation. Thus, this approximation optimizes the efficiency of each evaluation, without loss of accuracy.

Explicitly,


(3.8) $$P_{i}(z_{x},z_{y},t)=\int_{0}^{2\pi }\int_{0}^{r}\frac{1}{4\pi Dt}\exp\left(-\frac{(x_{i}+r^{\prime }\cos(\theta )-z_{x}{)}^{2}+(y_{i}+r^{\prime }\sin(\theta )-z_{y}{)}^{2}}{4Dt}\right)r^{\prime }\text{ d}r^{\prime }\text{ d}\theta ,$$

(3.9) $$\approx \frac{1}{4Dt}\exp\left(-\frac{(x_{i}-z_{x}{)}^{2}+(y_{i}-z_{y}{)}^{2}}{4Dt}\right)r^{2}={\tilde{P}}_{i}(z_{x},z_{y},t).$$

Following on from defining approximation[Disp-formula uFD10] 3.9, we can define the accompanying approximations ${\tilde{E}}_{i}$ and ${\tilde{F}}_{i}$ which have the same definitions as $E_{i}$ and $F_{i}$ , respectively, but ${\tilde{P}}_{i}$ is substituted for $P_{i}$ . Note that we still use Simpson’s rule for numerical integration [37,38] to calculate the approximate expected number of calls, ${\tilde{E}}_{i}$ , since there is no elementary indefinite integral for the function $e^{1/t}/t$ . However, we have reduced the problem of calculating $F_{i}$ from a three-dimensional integral to a one-dimensional integral approximation, ${\tilde{F}}_{i}$ , representing a time saving in the numerical evaluation.

**Figure 3.**
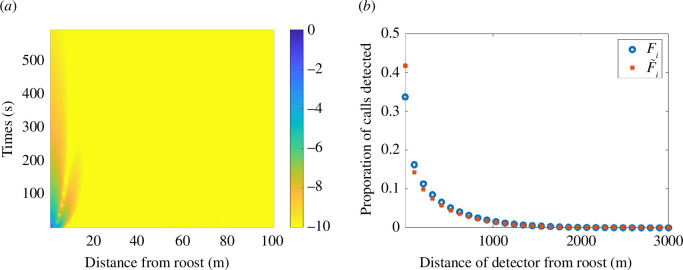
Illustrating the accuracy of integral approximation, equation (3.9). (*a*) Plot of $\log\left(|P_{i}-{\tilde{P}}_{i}|\right)$ , that is, the log absolute error between the full probability calculation (equation (3.8)), and the approximation (equation (3.9)). (*b*) Comparison between the expected proportion of calls and its approximation, $F_{i}$ and ${\tilde{F}}_{i}$ , using 30 detectors uniformly spaced between the roost and 3 km away from the roost. Parameters are $D=80\;m^{2}s^{-1}$ , $\left(z_{x},z_{y})=(0,0\right)$ and T=90 min.

The accuracy of our approximation is illustrated in figure 3, as we present the error between $P_{i}$ and ${\tilde{P}}_{i}$ , and between $F_{i}$ and ${\tilde{F}}_{i}$ . In calculating $P_{i}$ and ${\tilde{P}}_{i}$ , we let the detector location $\left(x_{i},y_{i}\right)$ range over all space, whereas in calculating $F_{i}$ and ${\tilde{F}}_{i}$ we choose specific detector locations, namely, we space 30 detectors uniformly along the x -axis in the interval [0,3000] m, explicitly, $(x_{i},y_{i})=(n3000/29,0),n=0,1,\ldots ,29$ .

Figure 3*a* demonstrates that the error in the probability approximation exponentially decreases away from the roost over time, as expected. Consequently, the comparison between $F_{i}$ and ${\tilde{F}}_{i}$ is excellent because the approximation is only poor for a small amount of time and over a small evolving location. Thus, over the time interval of 90 min the small error in the approximation to ${\tilde{P}}_{i}$ does not compound, resulting in uniformly excellent comparison that becomes better for detectors further away from the roost.

In this section, we have set up the basic mathematical framework which defines bat movement. Critically, we have been able to derive a function, ${\tilde{F}}_{i}$ , that, given a roost location, can be quickly estimated at all detector sites and provide a measure which can be compared with the call data from the surveys. In the next section, we will define a distance metric to compare the expected proportion of observations at each detector with data recorded from surveys and use this to estimate roost locations from survey data.

## 4. Estimating roost locations

To find potential roost locations, we define a distance metric, ρ , between the data and the expected observations. Let $\tilde{F}(z)=\left({\tilde{F}}_{1}(z),\ldots ,{\tilde{F}}_{N}(z)\right)$ be the vector of the expected proportion of calls at each detector (calculated from equation (3.7) assuming that the roost is at z and let $C=\left(C_{1},\ldots ,C_{N}\right)$ be the vector of the proportion of calls at each detector calculated from the data. We define


(4.1) $$\rho (C,\tilde{F}(z))=\frac{\sum_{i=1}^{N}({\tilde{F}}_{i}(z)-C_{i}{)}^{2}}{\max_{z}\left(\sum_{i=1}^{N}({\tilde{F}}_{i}(z)-C_{i}{)}^{2}\right)}.$$

Namely, ρ is the squared Euclidean distance between the expected proportion of observations at each detector and the survey data. This metric is then normalized by the maximum of all locations tested. Hence, ρ is bounded between 0 and 1, and low values of ρ should correlate with locations more likely to be the roost. Note that the squared distance is chosen here as opposed to, for example, the absolute difference, because the squared distance places more importance on the detectors which record high number of passes [39]; detectors which record particularly high numbers of bat passes are likely to be located close to the roost, while those which record fewer passes are likely to be located far from the roost. Moreover, tests were done varying the exponent and the regions of minimal ρ were generally not changed, although it was noted that an exponent of two minimized the average error over all locations, compared with an exponent of one or three (data not shown, but code available on GitHub, https://github.com/ThomasEWoolley/Bat_roost_detection).

In the following, we divide each location illustrated in figure 1*a* into a grid of 500×500 evenly spaced squares. The extent of the maps are defined by the maximum and minimum of the detectors' (x,y) coordinates.

**Figure 4.**
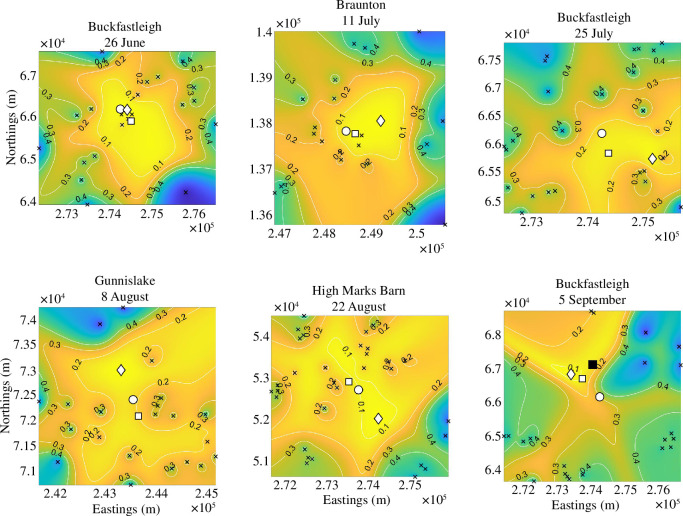
Plotting ρ using equation (4.1) over the survey site, which has been split into 500×500 equally spaced squares. The coloured background ranges from yellow when ρ=0 to blue when ρ=1 . The contours provide numerical values and aid visualization of the surface. The detectors are black crosses, the roost location, $z_{R}$ is a white circle, the estimated location, $z_{p}$ is a white diamond and the centre of calls is a white square. The black square in the Buckfastleigh 5 September subplot (bottom right) represents a more recently found satellite roost used by the same colony.


The resulting ρ surfaces are illustrated in figure 4. In all cases, the circular white marker is the known roost. The diamond marker is the minimum point of ρ , that is, the best point estimate our prediction provides. The square marker defines the geometric mean of the detectors weighted by the proportion of calls at each detector. We call this point the centre of calls, CC ,

(4.2) $$CC=\sum_{i=1}^{N}C_{i}X_{i}.$$

Note, we do not have to divide *CC* by the sum of the weights, $C_{i}$ , as they are proportions and, thus, by definition, sum to 1. Finally, the colour bar is the same across all maps and goes from dark blue ( ρ=1 ) to yellow ( ρ=0 ). Generally, the more yellow a region is the closer we should be to the roost. Table 2 provides distance values between the known roost, $z_{R}$ ; the centre of calls, CC and best point prediction, $z_{p}$ , such that $\rho (C,\tilde{F}(z_{p}))=\min(\rho )$ . These statistics offer information on the accuracy of the application of our roost-finding algorithm for each location in figure 4.

**Table 2.** Tabulation of the distances between the roost location, $z_{R}$ and the point estimates of $z_{p}$ (the best point prediction at min(ρ) ) and the centre of calls, CC as calculated using equations (4.1) and (4.2), respectively. All data are rounded to two decimal places.

date	roost	straight line distance in km between		
		$z_{R}$ and $z_{p}$	$z_{R}$ and CC	$z_{p}$ and CC
26 Jun 2016	Buckfastleigh	0.16	0.38	0.28
11 Jul 2016	Braunton	0.79	0.20	0.63
25 Jul 2016	Buckfastleigh	1.01	0.37	0.79
08 Aug 2016	Gunnislake	0.63	0.34	0.97
22 Aug 2016	High Marks Barn	0.84	0.31	1.14
05 Sep 2016	Buckfastleigh	1.08	0.74	0.36

Alongside the point estimates of CC and ρ we provide contour plots (white lines in figure 4) that demonstrate how ρ varies across the space. Critically, although the accuracy of a single-point estimate may be low, we can use the contour plots to provide optimal searchable regions, namely, we search the regions in order of increasing values of ρ . We define $\rho_{c}=\rho (C,\tilde{F}(z_{R}))$ , to be the value of ρ evaluated at the known roost point, $z_{R}$ . Table 3 provides the area that would need to be searched (as a percentage of the entire area mapped in figure 4) using the searching method of starting at the minimum of ρ and increasing the search area until we find the roost at $\rho_{c}$ .

**Table 3.** Percentage of areas below various thresholds of ρ along with the value of ρ at the roost, $\rho_{c}=\rho (C,\tilde{F}(z_{R}))$ . The percentage is relative to the size of the original searched area (see figure 4). All data are rounded to two decimal places.

date	roost	$\rho_{c}$	area covered by $\rho \leq \rho_{c}$ (km^2^)	percentage of mapped area bounded by			
				ρ≤0.1	ρ≤0.2	ρ≤0.3	$\rho \leq \rho_{c}$
26 Jun 2016	Buckfastleigh	0.04	0.04	16.98	36.06	68.11	0.28
11 Jul 2016	Braunton	0.06	0.99	11.15	43.49	71.48	6.36
25 Jul 2016	Buckfastleigh	0.17	0.86	0.00	17.78	47.51	9.00
8 Aug 2016	Gunnislake	0.18	3.08	0.00	36.45	74.95	25.19
22 Aug 2016	High Marks Barn	0.10	1.16	6.89	58.85	77.58	7.01
5 Sep 2016	Buckfastleigh	0.29	9.41	0.87	14.72	37.49	35.89

As a general overview, if we combine the information from figure 4 and tables 2 and 3, we observe that the predicted roosts’ locations appear to get worse over time. However, each survey time and location has its own nuances that need to be highlighted, thus, we now discuss each survey in turn and demonstrate how our data can be used to provide a picture regarding the success of the roost-finding algorithm.

For 26 June 2016 Buckfastleigh, the initial survey was conducted on an approximately 15 km^2^ area (see table 1). Table 2 informs us that the single-point prediction, $z_{p}$ , which predicts the location of the roost at $z_{R}$ does particularly well, being within 160 m of the actual roost. This predicted location is even better than the CC , which is 380 m away from the roost. Thus, if we were to start at the location of min(ρ) and search areas of increasing ρ until we found the roost then we would have to search 0.28% of the original survey area, or 0.04 km^2^. Thus, our roost-finding algorithm has saved over 99% of the searching effort required to locate the roost, should we not have known its location in advance.

In the case of the 11 July 2016 Braunton survey, the colony using the roost is much smaller than the Buckfastleigh roost (around one-third of the size) and, subsequently, the number of recorded calls within 90 min of sunset is lower. Additionally, as shown in figure 1*b*, the closest detectors to the roost are further than those for 26 June 2016 Buckfastleigh. However, even with all these weaknesses compared with 26 June 2016 Buckfastleigh, our method is highly effective in locating the roost, with an error of just 790 m, and once again reducing the search area by over 90%.

In contrast to the first Buckfastleigh survey, the second survey conducted at Buckfastleigh between 25 July and 3 August 2016, records more calls and has more detectors and a smaller initial search area. However, the algorithm provides a less successful point prediction as $z_{p}$ is 1.01 km away from the roost, whereas CC provides a more stable estimate as it is still less than half a kilometre away from the roost, similar to the first survey in Buckfastleigh. The reason the prediction is pulled southwest of the roost is because the biggest proportion of calls comes from a detector southwest of the roost (see figure 1*a*). The reason for the high number of passes is not immediately obvious from the data; however, when we look at the locations of hedgerows in the survey area (see figure 1*a*), we see that the detector was placed along one of the main hedgerow paths leading away from the centre of the survey area. As noted in §2, greater horseshoe bats are highly dependent on hedgerows for navigation and foraging, and it is likely that this particular hedgerow is used as a corridor between important parts of the landscape. In §6, we will suggest some possible adjustments to the model to account for the heterogeneity of the landscape.

Positively, even though the point estimate is worse than the first survey in Buckfastleigh, the amount of area that would need to be searched to find the roost is still very small. Although we have to search a bigger percentage of the area according to table 3 (9.00% compared with 0.28%) we are searching over a smaller area initially, and so, we would need to search 0.86 km^2^ in this second Buckfastleigh survey, compared with the area of 0.04 km^2^ in the first survey.

The colony using the roost on 8 August 2016 Gunnislake is much smaller than those using the other roosts, resulting in a much lower number of recordings. This means that stochastic effects will play more of a significant role in the prediction of this roost location. Such problems would be minimized by doing surveys over more days.

A further problem in the 8 August 2016 Gunnislake case is the flatness of ρ , as we observe much of the domain satisfies ρ<0.2 . The flatness of ρ means that many points are equally good and our algorithm cannot be more precise without additional data in the flat- ρ region. This problem could be solved by including more detectors in the ρ<0.2 region. More positively, our algorithm still saves over 74% in searching effort, as we would only have to search 3.08 km^2^ of the original space before we would be able to find the roost.

The ρ profile of 22 August 2016 High Marks Barn is similarly flat, because the area to the southeast of the roost is private land, and it was not possible to obtain the landowner’s permission to place detectors there. To compensate for the lack of homogeneity in the spatial distribution of the detectors, more detectors were used (more than any other survey). Thus, even with the heterogeneous detection space, we are able to save over 90% of the search effort.

The 5 September 2016 Buckfastleigh survey is the worst single-point prediction over all three Buckfastleigh surveys. Moreover, it is the worst single-point roost prediction out of all six surveys. Critically, although we would still save over 64% of our searching effort, the 5 September 2016 Buckfastleigh has the largest required search area, assuming that we start at the minimum of ρ and search areas with increasing values of ρ . Compared with the other two Buckfastleigh surveys there were hardly any detectors near to the roost with the average detector distance being approximately 2.5 km away from the roost (see figure 1*b*). The previous two Buckfastleigh surveys not only had detector location distributions with a smaller mean (1.5 km or less), but nearly all detectors were closer to the roost than 2.5 km. Moreover, the number of passes during this third Buckfastleigh survey was approximately the same as Gunnislake even though Buckfastleigh has over seven times as many bats recorded in its roost.

There are several possible reasons for the error in the estimate for the 5 September 2016 Buckfastleigh survey. Firstly, in 2018, a second large roost was found in a cave system close to the Abbey Inn, Buckfastleigh, around 1 km north of the main roost (shown as a black square in the 5 September 2016 Buckfastleigh of figure 4). In August 2018, counts were taken of greater horseshoe bats exiting this roost, and on each occasion, more than 800 bats were seen emerging. Our roost estimate $z_{p}$ is actually closer to this cave roost, being only 0.70 km away. Unfortunately, we cannot know if this roost was used in 2016 (or indeed if there is another, unknown, roost in the area); however, it is certainly possible that not all the bats recorded during this survey were from the main roost.

Critically, this second cave roost near Abbey Inn was first discovered in August, and may therefore only be used later in the season. Notably, the last Buckfastleigh survey was conducted in September, late in the season, and was the only survey at Buckfastleigh to be conducted after July. It is possible that by September, bats had already moved from the summer roost to the cave roost, or to another hibernation roost.

An alternative reason for the poor fitting of the September counts is that the maternity season is over, making way for the mating season and causing the nightly dispersal patterns to be more complicated. During the mating season, female bats go off in small groups to visit solitary males in mating roosts and, thus, sometimes they do not return to the main maternity roost as they stay with the males [40].

**Figure 5.**
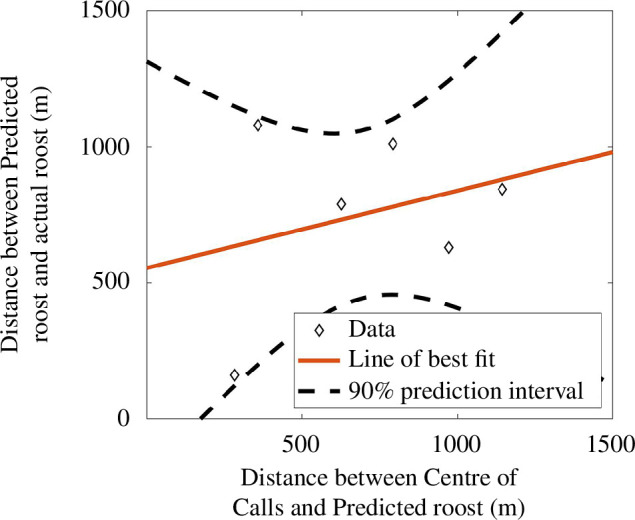
Correlating the error in the predicted distance, $|z_{R}-z_{p}|$ , with the distance between the two point estimates of the predicted roost and centre of calls, $|CC-z_{p}|$ .

Finally, as a means of providing confidence in our point predictions, we note that as the distance between the actual and predicted roost becomes worse, the distance between $z_{p}$ and CC also increases. Figure 5 illustrates the correlation between the distance between the predicted location $z_{p}$ and CC , and the distance between the predicted location $z_{p}$ and $z_{R}$ . Thus, using figure 5, if we were to apply our algorithm to a new set of data where the roost was not known, then our confidence in the location of min(ρ) being near the roost can be governed by how far min(ρ) is from CC . For example, as a rule of thumb, if the locations of $z_{p}$ and CC are separated by a few hundred metres then we would expect the roost to be within a few hundred metres of CC and $z_{p}$ , while if CC and $z_{p}$ are separated by a kilometre, or more, then it is likely that the point prediction is performing more poorly although the area searching algorithm should still lead to large savings in the effort.

## 5. Roosts outside the detector coverage

In the six cases we are considering here, the roost location was either known or suspected. Thus, microphones could be spread in all directions around the suspected roost, meaning that the geometric mean of the detectors was likely to be a good estimate for the roost location regardless of any further call data. This is why the roost in each of the cases of figure 4 is close to the centre of the image. This raises some limitations of CC . Firstly, the ability of CC to provide a good estimation for the roost location may depend on the prior knowledge of the ecological surveyor. Secondly, CC can only estimate points within the spatial limits of the detectors. Thus, in the case that (i) we do not where a roost is and (ii) the roost is outside of our detector placement area, CC cannot provide a good point estimate for the roost location. The best CC could do is be close to one of the boundaries, indicating that the roost is unlikely to have been contained within the initially surveyed area.

The construction of the ρ surface does not have any of these limitations. Thus, in this section, we approach the question of how good our algorithm is at identifying a roost outside of the detector locations in two ways.

Firstly, we use a stochastic agent-based model of bat movement and simulate the call distributions of a roost outside of the 26 June 2016 Buckfastleigh detector distribution (refer to appendix A). Secondly, because the 22 August 2016 High Marks Barn survey had the largest number of detectors, we simply rerun our analysis on High Marks Barn, but without including data from all detectors to the south of the roost. Another reason for using High Marks Barn is because of the restriction that the area to the southwest was private and detectors could not be placed there. Thus, we seek to consider a worst-case scenario that a roost is on private land and no detectors can be placed on one side of the roost.

The reason we ran these two experiments is because the first approach could be criticized for being too artificial. Namely, although our previous research [18,19] has shown that the movement features of the greater horseshoe bat population can be characterized as diffusive (at least for the first 90 min after sunset), we would be using a diffusion-based search model to find simulated diffusive agents that would not be reading the domain as a real bat would. Thus, the first test provides a proof-of-concept demonstration that our algorithm can find a roost outside the extent of a microphone placement, using a realistic set of detector distributions.

The second experiment, although more realistic, is weakened because we are removing approximately half of the data from the space. Specifically, we will use 193 call recordings from the 18 detectors north of the roost (originally, we used 272 call recordings from 31 detectors; see table 1). Thus, it should be kept in mind that this reduction in data will influence how well ρ can predict the roost location.

**Figure 6.**
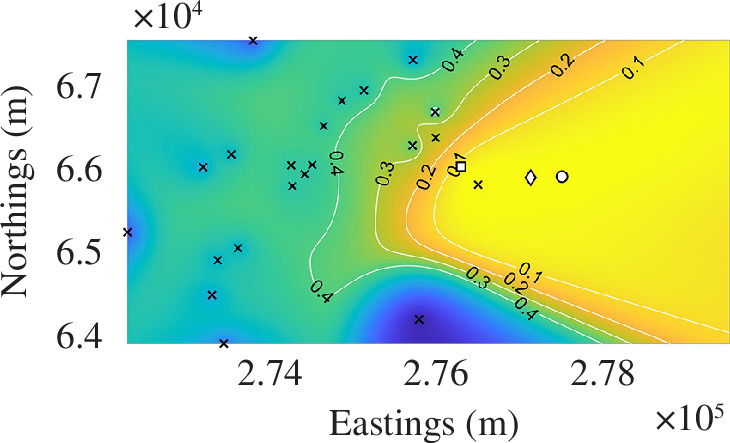
Predicting the location of a roost outside of a detector distribution using simulated data. The coloured background represents ρ calculated using equation (4.1) and ranges from 0 (yellow) to 1 (blue). The contours provide numerical values and aid visualization of the surface. The detector locations are the same as in the 26 June 2016 Buckfastleigh survey and are represented as black crosses, the simulated roost location is a white circle, the estimated location is a white diamond and the centre of calls is a white square. The bat flight trajectories were simulated using equation (A.1). The parameter value is D=81.7 m^2 ^s^−1^.

The result of our first experiment in finding a roost outside of the detector placement is shown in figure 6. Using the detector placements as defined in the 26 June 2016 Buckfastleigh survey, we simulated 600 bats starting at a roost 1 km east of the furthest east detector. The agents move diffusively, with a diffusion rate of D=81.7 m^2^ s^−1^ [19]. The search space has been extended by 3 km to the east from the original search space (refer to figure 1 *a*), so the search space is now 25.98 km^2^, compared with the original search space of 15.13 km^2^.

Figure 6 illustrates the problem stated above; the CC point (white square) is bound to lie within the extent of the detector placement. Thus, the distance error between the roost (white circle) $z_{p}$ and CC can be made arbitrarily large. Moreover, the predicted location of min(*ρ*) (white diamond) is only 375 m from the simulated roost and we would only have to search 0.3% of the domain (or 0.78 km^2^) to find the roost. Thus, overall, we can see that our searching algorithm is successful in finding the roost outside of the detector domain in the case of simulated bat movement data.

**Figure 7.**
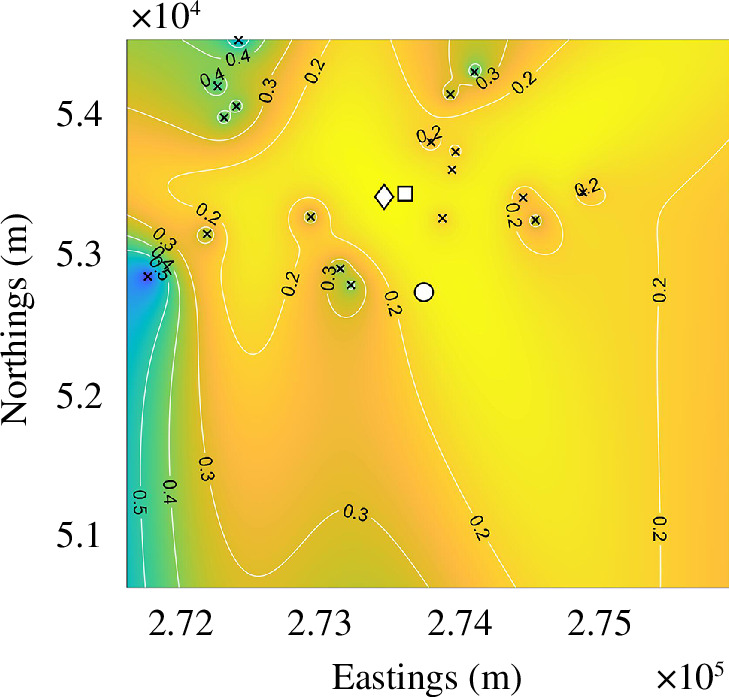
Predicting the location of a roost outside of a detector distribution using only data to the north of the detector from the 22 August 2016 High Marks Barn survey. The coloured background represents ρ and ranges from 0 to 1. The contours provide numerical values and aid visualization of the surface. The detector locations are represented as black crosses, the roost location is a white circle, the estimated location is a white diamond and the centre of calls is a white square.

In the second experiment, illustrated in figure 7, both $z_{p}$ and CC are placed fairly central within all the detectors and are approximately 700 m away from the roost. However, although both point estimates are equally mistaken, the ρ is able to highlight the areas that are equally likely to hold the roost. Clearly the majority of the low ρ region lies much further to the south of the detector region, indicating that we are looking in the wrong region.

Of course, the prediction surface generated in figure 6 is based on a lot less information than we would normally expect, thus, we should not be surprised that neither point prediction is good. In such cases, we would recommend the survey be rerun with the detectors placed (if possible) further to the southeast using the information that can be gleaned from figure 7. This should cause more detectors to be placed closer to the original roost leading to a better resulting prediction.

## 6. Discussion

In this article, we have combined data from static audio detectors and mathematical modelling to create an efficient algorithm for identifying possible bat roost locations, which is currently a highly labour-intensive process. Critically, even if a single-point estimate is not accurate, our spatial metric provides an optimized means of searching space that can reduce our searching effort by over 90% at best and 50% at worst. Before we draw overall conclusions, we discuss some of the complicating factors that would necessarily limit the accuracy of our current algorithm, and offer jumping-off points for further refinements.

Table 2 demonstrates that the point estimate CC is consistently as good as, if not better than, our diffusion-based movement algorithm at predicting a roost’s location. However, although CC is a useful additional measure, it does have a number of drawbacks that would means its sole usage would be problematic.

Firstly, CC is a single-point estimate, whereas ρ provides an entire likelihood surface. Thus, it is harder to supply any confidence bounds on CC . In particular, it is harder to predict when CC will provide a poor performance. In contrast, we saw that ρ was a fairly flat surface in the case of High Marks Barn, and, thus, we would immediately be able to deduce that there would be many equally good potential locations, meaning that $z_{p}$ was less likely to be accurate.

Secondly, CC cannot be generalized beyond being a single-point estimate to include potentially multiple roost sources. Multiple sources can easily be included in the definition of ρ as we would simply include more Gaussian distributions in the definition of equation (3.8). Of course, including multiple sources would lead to a combinatorial slowing of the algorithm, as all combinations of points would need to be evaluated, but it would be possible, to find the best two or three locations for potential roosts.

Thirdly, as mentioned in §5, CC is bound to lie within the extent of the detector placement. Our prediction method has no such restrictions. Combining these insights with the correlation between the accuracy of our prediction and distance from CC we can begin to suggest whether we are looking in the right place to begin with.

We now consider factors that would have influenced the accuracy of our prediction stemming from the data. All six surveys were conducted at maternity roosts, during the summer months. Maternity roosts are populated by females and their young, while mature males generally use separate roosting sites and therefore do not disperse from the maternity roost [41]. However, it is not possible to distinguish between the calls of males and females, and therefore calls from males are recorded in the same way, adding noise to the number of passes at each detector. If some detectors are placed particularly close to male roosting sites, this could skew the number of passes recorded and therefore negatively affect the roost estimate.

Additionally, in each survey case, we have only been looking for one roost, but it is always possible that there are multiple roosts in the area, as was discovered later in Buckfastleigh. Extending our current method to include more sources is fairly trivial. However, in the present case, such complexities would add to the overall difficulty of pinpointing a single ‘best’ location.

Environmental conditions, although not considered here, could play a significant role. For simplicity, we have assumed that each night bats disperse from the roost after sunset regardless of the temperature and weather conditions that night. However, bats expend much of their energy to maintain stable body temperatures and are sensitive to climatic conditions [42]. Thus, inclement weather is likely to change foraging behaviour.

We should also note how bats use their roosts and how the landscape changes throughout the year. During the summer, females use maternity roosts in which they give birth and raise their young. Maternity colonies generally form in early summer, from May onwards. While greater horseshoes in southwest England tend to give birth between late June and July [41], birth timing can vary wildly depending on the temperatures throughout spring and early summer. Mothers care for their pups for around eight weeks before they are weaned and ready to forage independently, probably leading to changes in foraging behaviour.

As mating in greater horseshoes occurs from late August onwards, bats tend to travel further in search of mates, potentially abandoning maternity roosts to move closer to mating sites [43]. Thus, the diffusion model used at the start of the maternity season in May–June may not be generalizable to the end of the maternity season in late August–September, or at least it may require a different diffusion rate.

Evidence for this can be seen in table 1, where the number of detections during the final Buckfastleigh survey (which occurs in September) is significantly lower than the previous Buckfastleigh surveys, suggesting the possible abandonment of the maternity roost by some adult females in favour of mating sites.

The error in the roost estimate for each survey is shown in tables 2 and 3. Notably, errors are higher in surveys conducted towards the end of summer compared with those in June or July, although with only six surveys it is difficult to generalize these results. A second radio-tracking survey later in the season would be extremely useful to verify if bats still move via diffusion, and if so, if the diffusion coefficient varies throughout the summer. However, each radio-tracking survey requires hundreds of person hours and is therefore beyond the scope of this article.

As noted in §2, these six surveys were conducted to study landscape use around the roost [28]. Detectors were deliberately placed to cover a variety of terrain types, some of which (e.g. hedgerows, where insects are abundant and there is a cover from predators) are known to be favourable for bat movement and some of which are detrimental to bat movement (e.g. busy roads, where there are bright lights, loud noises and few insects to feed on). The terrain in the four locations is heterogeneous, including a mixture of rural farmland and urban areas. It is almost certain therefore that the number of passes recorded at each detector has been affected by the surrounding terrain [44]. Future work will look to enhance our roost prediction accuracy by incorporating landscape data into the model.

Although extending equation (3.1) to include anisotropic diffusive movement is possible, as we would just include a spatial component in D , we would not be able to provide a complete solution in the form of equation (3.3), thus, we would need to rely more heavily on simulation, which would greatly increase the complexity of the method. Further, being able to define an anisotropic diffusion function based on landscape topography is not trivial: the data for different landscape features is collected by different organizations (e.g. the locations of roads and buildings can be extracted from Ordnance Survey maps, while data such as the location of streetlights is often only available on request from Local Authorities). Moreover, the coverage of this data is not complete. Thus, having only partial data coverage in some locations may end up biasing the results more than not have any additional data anywhere. Finally, much of this data is only available through subscription services that an ecologist searching for a roost may not necessarily have access to.

A straightforward way to reduce the effect of landscape effects would be to place all detectors in favourable terrain, for example, only on hedgerows. This could have the added benefit of increasing the expected number of recordings at each detector, thereby increasing the quantity of data recorded. However, the number of passes recorded at each detector is affected not just by the surrounding landscape, but also by the functional connectivity between the roost and the detector: bats are unlikely to cross motorways each night to reach a hedgerow, regardless of how many insects are available in the hedgerow. To further improve the validity of the diffusion model, we would need to consider the complex effects of landscape connectivity on bat movement [45].

In addition to variations in bat behaviour, imperfections in data collection during surveys are also probable. Detectors are typically placed outdoors, often in locations with public right of way or on farmland, making them susceptible to theft, vandalism and damage caused by animals. Consequently, some detectors may fail before the full 7 days survey concludes. Such imperfections can give us a misleading view of the environment. Although we have averaged over the number of days to provide a daily count, detectors with shorter operational durations due to malfunctions may introduce higher errors in the calculation of ρ . Such errors may account for the low accuracy in the roost prediction for High Marks Barn, as these detectors exhibited the lowest average survival time span (see figure 1*c*).

A further imperfection in the methods comes from the use of continuous and deterministic models to describe inherently discrete and stochastic behaviour. At higher distances from the roost, stochasticity in movement plays a larger part in the number of passes recorded. Additionally, we note that the colony using the Buckfastleigh roost is significantly larger than an average colony, and the importance of considering stochastic effects would increase with smaller numbers of bats [46,47].

This stochasticity could, in part, explain the poor roost prediction in the last Buckfastleigh survey. Namely, all the detectors in this survey were placed, on average, further away from the roost than in any other survey (see figure 1*b*). Adding detectors closer to the true roost could certainly improve the estimate. However, in a roost-finding survey, the location of the true roost would not be known and we therefore would not know how to place detectors near to it.

A more accurate method would account for landscape effects, time of year, solitary males, weather conditions, stochasticity, roost size and survey design. However, incorporating each new variable increases the model’s complexity. Without acquiring substantial quantities of new data, which would be both time-consuming and costly, studying the interdependence of these variables becomes challenging.

Speed of computation being less than a day is particularly important for our plans for future work. Building on these results, we want to begin to investigate how detector arrays could be updated (potentially daily) to optimally survey space and, thus, provide increased accuracy in locating bat roosts. Specifically, we aim to improve survey design by using an iterative Bayesian global optimization approach to ensure that the data recorded is the most useful for estimating roost locations.

Overall, we state that the benefits of using our algorithm to find roosts far outweigh its limitations. In all cases, we have been able to reduce the potential search space by at least 64%. Moreover, it should be remembered that our diffusion algorithm was not parametrized on any of the data contained within this article; rather, we used diffusion coefficients derived from radio-tracking surveys conducted at a different time and location [18,19]. The fact that these parameters and motion characteristics yielded the accuracy we achieved provides confidence in the generality of our approach (at least for greater horseshoe bats).

We only used the first 90 minutes after sunset of microphone data, as previous work has shown that a simple diffusive model can recreate the bat’s motion features. However, we have been able to model not just the initial bat spread, but also the later ‘return to roost’ phase [18,19]. Using these models we could increase our accuracy (at the expense of complexity and speed) by extending this work to use the full nights' worth of call data.

Finally, even in the worst-case scenario of having to search 36% of the space, this is without considering the expertise of the ecological researchers performing the survey. Ecologists have a wealth of practical knowledge and experience regarding potential roosting locations. Thus, overlaying potential location ρ maps onto terrain feature maps allows ecologists to prioritize search areas within these suggested regions, optimizing our searching algorithm further by combining digital and practical expertise.

## Data Availability

Data and relevant code for this research work are stored in GitHub: https://github.com/ThomasEWoolley/Bat_roost_ detection and have been archived within the Zenodo repository [51].

## Appendix A. Simulated data

In §5, we simulate call data from a bat roost outside of the 26 June 2016 Buckfastleigh detector placement region. To do this, we simulate 600 diffusive trajectories using a Wiener process [48–50] centred at the new roost location (277 512, 65 944) m. The simulated roost location was chosen to be 1 km further east than the most easterly detector and placed vertically at the mean y value position taken overall detector locations (see figure 6). We simulate 600 agents as this is the approximate number of recordings taken during the 26 June 2016 Buckfastleigh survey.

The Wiener process simulates agents undergoing random walks that reproduces statistics that matches the formal definition of Brownian motion, or diffusion. The location, $\left(b_{x}(t),b_{y}(t)\right)$ , of an individual agent at time t is defined by

(A.1)
$$\left(\begin{array}{c} b_{x}\,\left(t+\Delta \,t\right)\\ b_{y}\,\left(t+\Delta \,t\right) \end{array}\right)=\left(\begin{array}{c} b_{x}\,\left(t\right)\\ b_{y}\,\left(t\right) \end{array}\right)+\sqrt{2\,D\,\Delta \,t}\,\left(\begin{array}{c} r_{x}\\ r_{y} \end{array}\right)$$

where D= 81.7 m^2^ s^−1^ is the diffusion rate of greater horseshoe bats, as found in Henley *et al*. [19], $r_{x}$ and $r_{y}$ are two randomly generated numbers sampled from a normal distribution with mean 0 and standard deviation 1, N(0,1) , and Δt=1 s is the time step between observations. We use the roost location as the initial condition for all bats.

Using equation (A.1), we simulate 600 trajectories over a 90 min period. We then count how many times a trajectory was within a circle of radius 15 m around each of the detector locations from the 26 June 2016 Buckfastleigh survey. This data is used to form the ρ surface shown in figure 6.
